# A Study of l-Lysine-Stabilized Iron Oxide Nanoparticles (IONPs) on Microalgae Biofilm Formation of *Chlorella vulgaris*

**DOI:** 10.1007/s12033-022-00454-8

**Published:** 2022-01-31

**Authors:** Seyedeh-Masoumeh Taghizadeh, Alireza Ebrahiminezhad, Mohammad Javad Raee, Hamidreza Ramezani, Aydin Berenjian, Younes Ghasemi

**Affiliations:** 1 Biotechnology Research Center, Shiraz University of Medical Sciences, Shiraz, Iran; 2 Center for Nanotechnology in Drug Delivery, Shiraz University of Medical Sciences, Shiraz, Iran; 3 Department of Pharmaceutical Biotechnology, School of Pharmacy and Pharmaceutical Sciences Research Centre, Shiraz University of Medical Sciences, Shiraz, Iran; 4 School of Engineering, Faculty of Science and Engineering, the University of Waikato, Hamilton, 3240 New Zealand; 5 Department of Agricultural and Biological Engineering, Pennsylvania State University, 221 Agricultural Engineering Building, University Park, PA 16802 USA

**Keywords:** Aquatic environments, Crystal violet, Magnetic nanoparticles, Fe_3_O_4_, Magnetite nanoparticles

## Abstract

Despite iron-based nanoparticles gaining huge attraction in various field of sciences and technology, their application rises ecological concerns due to lack of studies on their interaction with microbial cells populations and communities, such as biofilms. In this study, *Chlorella vulgaris* cells were employed as a model of aquatic microalgae to investigate the impacts of l-lysine-coated iron oxide nanoparticles (lys@IONPs) on microalgal growth and biofilm formation. In this regard, *C. vulgaris* cells were exposed to different concentrations of lys@IONPs and the growth of cells was evaluated by OD600 and biofilm formation was analyzed using crystal violet staining throughout 12 days. It was revealed that low concentration of nanoparticles (< 400 µg/mL) can promote cell growth and biofilm formation. However, higher concentrations have an adverse effect on microalgal communities. It is interesting that microalgal growth and biofilm are concentration- and exposure time-dependent to lys@IONPs. Over long period (~ 12 days) exposure to high concentrations of nanoparticles, cells can adapt with the condition, so growth was raised and biofilm started to develop. Results of the present study could be considered in ecological issues and also bioprocesses using microalgal cells.

## Introduction

Iron-based nanoparticles are one of the most applied nanostructures in various fields of sciences and technologies. Due to their unique physicochemical properties these particles have gained applications in various fields science and engineering [1–6]. Nowadays iron oxide nanoparticles are employed in medical industries for magnetic resonance imaging (MRI), magnetic particle imaging (MPI), drug delivery, and hyperthermia [7].

According to introduction of iron nanoparticles to the life sciences, many studies have been done to discover the interaction of living cells with these nanostructures [8, 9]. Concerning these investigations, it was revealed that iron nanoparticles have a concentration-dependent effect on the cells. At low concentrations, nanoparticles can act as a source of iron to provide valuable iron ions and promote cell growth. By increase in the concentration of nanoparticles, toxic effects are increasingly prevailing and reduction in the cell growth and viability would be observed [9, 10].

In addition to cell growth rate, iron nanoparticles can affect cell communities such as biofilms due to their impacts on the cell surface feature. In some cases, exposure of microalgae to iron nanoparticles can significantly reduce biofilm formation due to its ability to attach to cell surface via non-specific interactions, such as hydrophobic, electrostatic, and Van der Waals force [2, 11–18]. Decoration of microbial cells with iron nanoparticles has some effects on the cell’s adhesion capability. Iron nanoparticles can block adhesion factors on the microbial cells surface, hence can reduce the cells adhesion power and biofilm formation [8, 11, 19]. This effect was observed by both Gram-negative (*Klebsiella pneumoniae*, *Escherichia coli*, *Pseudomonas aeruginosa*, *Listeria monocytogenes*) and Gram-positive (*Staphylococus aureus*, *Entereococcus faecalis*, *Bacillus subtilis*) bacterial cells as well as yeast (*Candida albicans*) cells [8, 11, 19].

Iron nanoparticles have also immense impact on the growth and metabolic states of aquatic micro-organisms, such as microalgae [20]. These cells are located in the base of ecological pyramid and play a key role in the ecological systems. They are valuable sources of nutrients and bioactive compounds such as carbohydrates, proteins, lipids, omega-3 fatty acids, vitamins, antioxidants, pigments, minerals, and other essential components that gained application in nutritional and pharmaceutical products [21]. Microalgae like many micro-organisms exhibit two different modes of growth, the planktonic mode (free-living cells) and the biofilm mode. In planktonic mode the micro-organisms are in single cell form and can be floating in the culture media. But, in biofilm mode the single cells of micro-organisms attach together with some self-produced matrixes and form the structured community. These communities can tolerate the harsh condition, such as starvation, dehydration, and presence of antimicrobial agents. Biofilm communities could be formed on the surface of all materials that expose to micro-organisms [22]. In the biodiesel production, increase in microalgal biofilm can be beneficial due to increase in biomass productivities, water management, and facile harvest of cells from culture media [23]. Biofilm formation in industrial processes on the surfaces of devices leads to severe consequences, such as decrease in heat and nutrient transfer efficacy and mechanical clogging in submerged systems [22].

Developments in industrial application of iron nanoparticles rise the concern about their possible ecological side effects especially biofilm formation. So it seems necessary to evaluate their impact on all aspects of aquatic life. As indicated, various reports are available about the impact of iron nanoparticles on the physiology of microalgal cells [20]. But there are no data about the effects of iron nanoparticles on the formation of microalgal multicellular structures, such as biofilms. In this study, *Chlorella vulgaris* was employed as a model of aquatic microalgae to investigate the impact of iron nanoparticles on microalgal biofilm formation. In this regard, *C. vulgaris* cells were exposed to different concentrations of iron nanoparticles and biofilm formation was evaluated.

## Materials and Methods

### Materials

All chemicals were purchased from Merck (Darmstadt, Germany) in analytical grade and used without any further purification. Millipore water (resistance > 18 MΩ/cm) was used throughout the experiment.

### Synthesis of l-Lysine-Coated Iron Oxide Nanoparticles

**Scheme 1:**
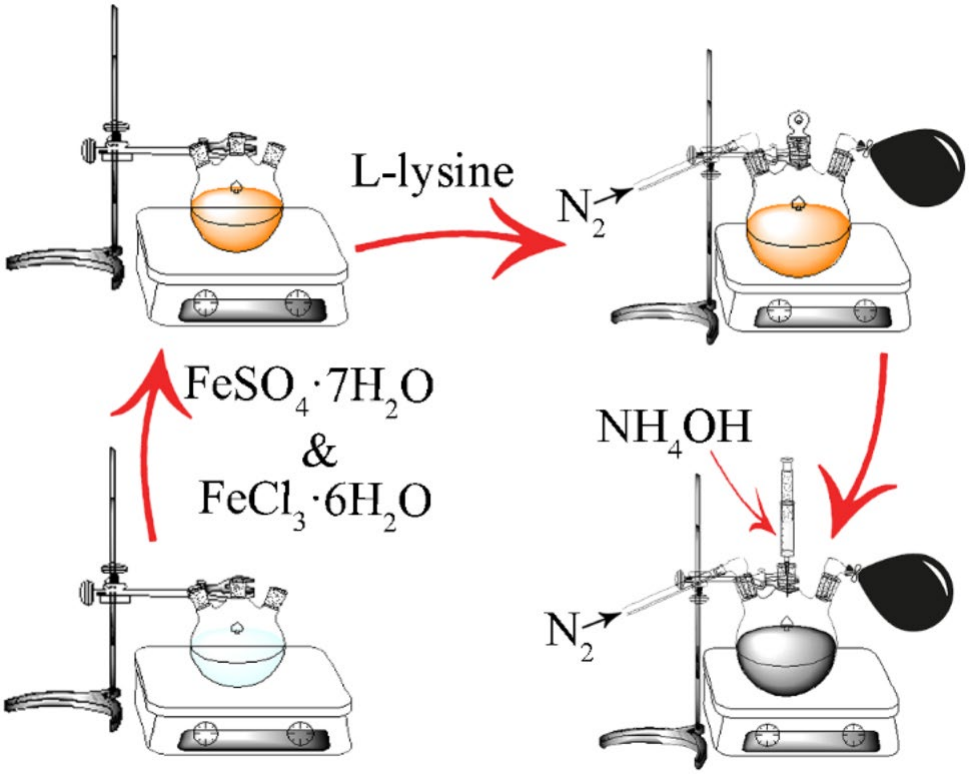
Schematic illustration of lys@IONPs synthesis

l-Lysine-coated iron oxide nanoparticles (lys@IONPs) were synthesized via a co-precipitation reaction. The reaction was carried out in the presence of l-lysine to provide nanoparticles with amino acid coating and increased stability [24]. The reaction was conducted in aqueous solution under nitrogen protection. In brief, 0.74 g of ferrous sulfate heptahydrate (FeSO_4_·7H_2_O) and 1.17 g of ferric chloride hexahydrate (FeCl_3_·6H_2_O) (1.75:1 ratio) were dissolved in 45 mL deionized water and magnetically stirred for 30 min at 70 °C. Afterward, l-lysine solution (5 mL, 32%) was added and stirring was continued for another 30 min. The nanoparticles synthesis was started by injection of 6.5 mL ammonium hydroxide (NH_4_OH, 25%) to the solution and reaction was continued by magnetic stirring using a magnet bar and hitter stirrer for 1.5 h (Scheme 1). The resultant precipitate (lys@IONPs) was separated from the reaction by centrifugation (4000 rpm, 20 min) and washed three times with water. The resultant lys@IONPs was dried in an oven at 50 °C.

Naked iron oxide nanoparticles without l-lysine amino acids from our previous work were employed as a control material for demonstration of lys@IONPs synthesis [20].

### Characterization of Iron Oxide Nanoparticles

Morphological appearance of resulted naked IONPs and lys@IONPs was characterized using transmission electron microscopy (TEM, Zeiss EM10C, HT 80 kV, Germany). Particles size distribution was analyzed using ImageJ software version 1.47v (available at https://imagej.nih.gov/ij/) by calculating average size of 100 particles of three different samples [25]. Crystal structure of the synthesized nanoparticles was analyzed by X-ray powder diffractometry (XRD, Siemens D5000, Germany) using fine powder of prepared nanoparticles. Fourier transform infrared (FTIR, Perkin-Elmer, Germany) spectroscopy was employed to study the chemical structure and functional groups on the naked IONPs and lys@IONPs. Magnetic properties of the particles were measured by vibrating sample magnetometer (VSM, Meghnatis Daghigh Kavir Co., Iran).

### Interaction of Nanoparticles with Microalgal Cells

A fresh culture of *C. vulgaris* cells (5 × 10^6^ cell/mL) was prepared in BG-11 broth medium and the cells were exposed to lys@IONPs in various concentrations (0, 12.5, 25, 50, 100, 200, 400, 600, and 800 μg/mL) in 10 mL vials. Vials were incubated in a microalgal culture room at 27 °C that was set to provide 16-h light/8-h dark cycle with light intensity of 60 μE/m/s using cool white fluorescent lamps.

Interactions of lys@IONPs with the surface of microalgal cells was visualized using field emission scanning electron microscopy (FESEM, MIRA3 TESCAN-XMU, TESCAN, Czech). Sample preparations were done based on our previous report using glutaraldehyde to fix the cells and increasing concentrations of ethanol for cells dehydration [20].

### Growth Assay

Impacts of synthesized nanoparticles on the growth of *C. vulgaris* cells were evaluated by measurements of optical density at 600 nm (OD600) using an Eppendorf Biophotometer® plus (Hamburg, Germany).

### Biofilm Assay

Formation of microbial community as biofilm was evaluated using crystal violet staining. After each predetermined exposure time, the submerged cells were dumped out and biofilm was rinsed with normal saline for three times to remove free cells. Biofilm was stained by crystal violet (3 mL, 0.1%) for 15 min. Vials were washed with normal saline and dried overnight at ambient atmosphere. Stained biofilm was exposed to acetic acid (30%, 1 mL) and incubated at room temperature for 15 min to solubilize the film. Resulting homogeneous solution (100 μL) was transferred to a 96-well plate and absorbance was measured at 550 nm against acetic acid (30%) as blank [11, 26].

### Statistical Analysis

All experiments were done in triplicates and statistical analysis were performed using IBM SPSS statistics for Windows (version 20, USA). Analysis of variance (ANOVA) and post hoc comparisons were made by Dunnett’s test. The *p* value < 0.05 was considered as significant difference.

## Results and Discussion

### Characterization of Iron Oxide Nanoparticles

**Fig. 1:**
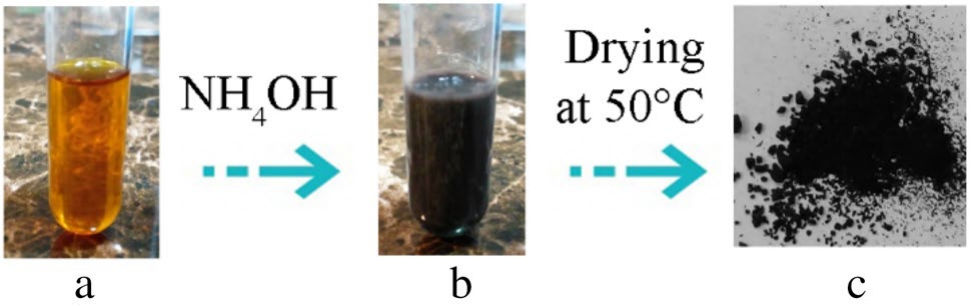
Color change during synthesis of lys@IONPs, before reaction (**a**), after reaction (**b**), and resultant powder after drying (**c**)

By injection of ammonium hydroxide in the solution of iron ions a sudden change in the color from yellow–orange to dark black was observed which was due to formation of magnetite nuclei (Fig. 1). A 1.5-h reaction time was considered for the growth step to provide particles with nanodiameter range [7, 24].

**Fig. 2:**
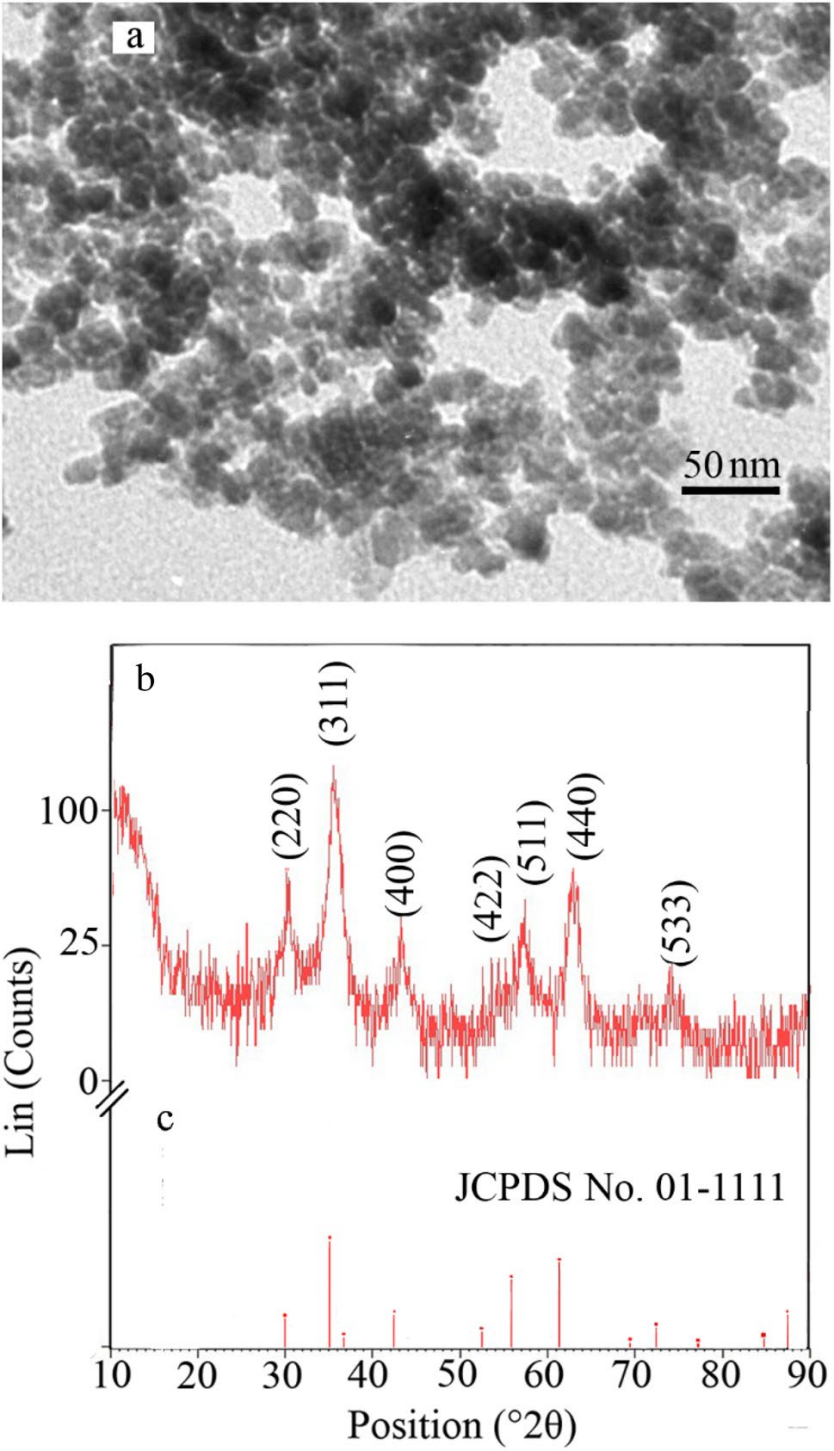
TEM micrographs of the synthesized iron nanoparticles with spherical appearance (**a**), X-ray diffraction pattern of synthesized l-lys@IONPs (**b**), and the pattern of reference magnetite (**c**)

Visual appearance of the resulted lys@IONPs is illustrated in a TEM micrograph as shown in Fig. 2a. Nanoparticles were spherical in shape. Crystalline structure of prepared particles was evaluated by XRD analysis and diffraction peaks were appeared at 30°, 35.5°, 43°, 57°, and 63° of 2*θ* value (Fig. 2b). The recorded pattern was in accordance with characteristic feature of magnetite (Fe_3_O_4_) crystals (Card NO. 96-900-5839). It was demonstrated that the core of magnetite nanoparticles was synthesized during synthesis process. The background of XRD pattern noise could be due to amorphous coating with l-lysine amino acid. Previous investigations reported the noisy background in coating of magnetite nanoparticles with amorphous materials, such as fucan [27]. Presence of amorphous materials in the resultant lys@IONPs was demonstrated with a hump in the range of 10–20° of 2*θ* value, which was reported previously [28].

**Fig. 3:**
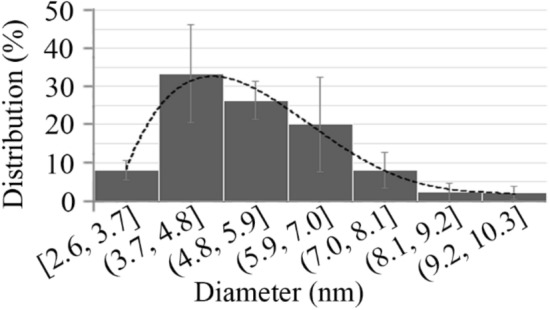
Size distribution of iron nanoparticles calculated by measuring the diameter of 100 particles (in three different samples)

Figure 3 illustrates the particle size distribution based on TEM micrographs. TEM micrograph showed particles with a narrow particle size distribution. Nanoparticles were measured to have a diameter ranging from 2.6 to 10.3 nm with an average size of 6 nm.

**Fig. 4:**
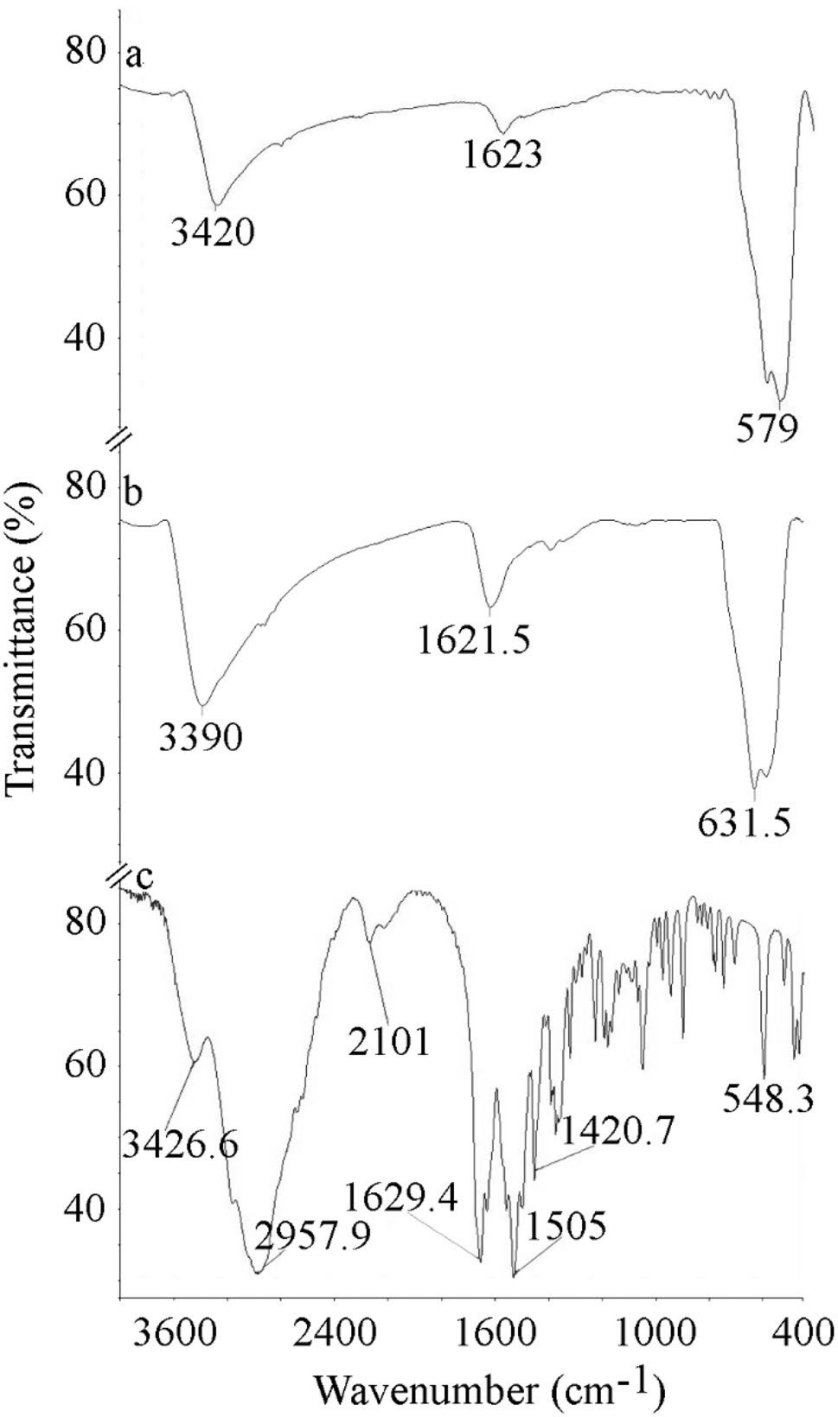
FTIR spectra of naked IONPs (a), synthesized lys@IONPs (b), and l-lysine amino acid (c)

FTIR graphs of naked IONPs and lys@IONPs are shown in Fig. 4a and b, respectively. The naked IONPs and lys@IONPs depicted characteristic peaks of Fe–O bonds at 579 cm^−1^ and 631.5 cm^−1^, respectively. Absorption peaks due to stretching vibration of hydroxyl groups on the surface of synthesized nanoparticles were observed at 3420 cm^−1^ in IONPs and at 3390 cm^−1^ in lys@IONPs. Peaks due to deforming vibration of hydroxyl groups were occurred at 1623 cm^−1^ and 1621.5 cm^−1^ in naked IONPs and lys@IONPs, respectively. The shift of OH spectra to lower wavenumbers can be due to overlap between hydroxyl group on surface of IONPs and amino group vibration from lysine coating. FTIR spectra of l-lysine amino acid are illustrated in Fig. 4c. Compared to pure l-lysine, shortening of the carboxyl group’s peak could be resulted from the interaction with OH groups on the surface of IONPs. Previous studies suggested that amino acids potentially interact with iron nanoparticles from their carboxyl groups and functional groups on the side chains [24].

**Fig. 5:**
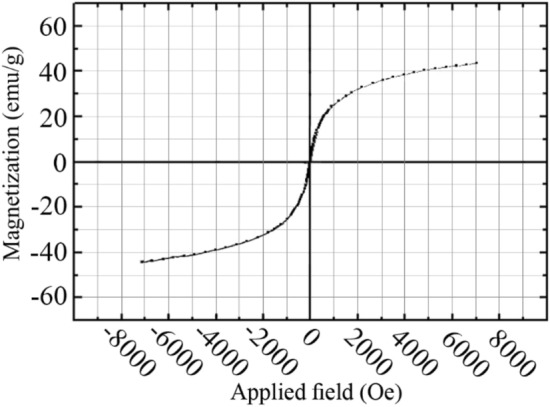
VSM graph of synthesized iron nanoparticles recorded at room temperature

Magnetic evaluations revealed super-paramagnetic nature of nanoparticles. The VSM graph was completely reversible and no remnant was observed that is characteristic of super-paramagnetic nanoparticles (Fig. 5). Prepared nanoparticles showed a saturation magnetization equal to 42 emu/g. This value was in close agreement with previous reports for amino acid-coated magnetite nanoparticles [29, 30]. In contrast to other biocompatible coatings, amino acids can be considered as one of the promising coatings with lowest adverse effects on the magnetic properties of the magnetite nanoparticles [31, 32].

### Interaction of Nanoparticles with Microalgal Cells

**Fig. 6:**
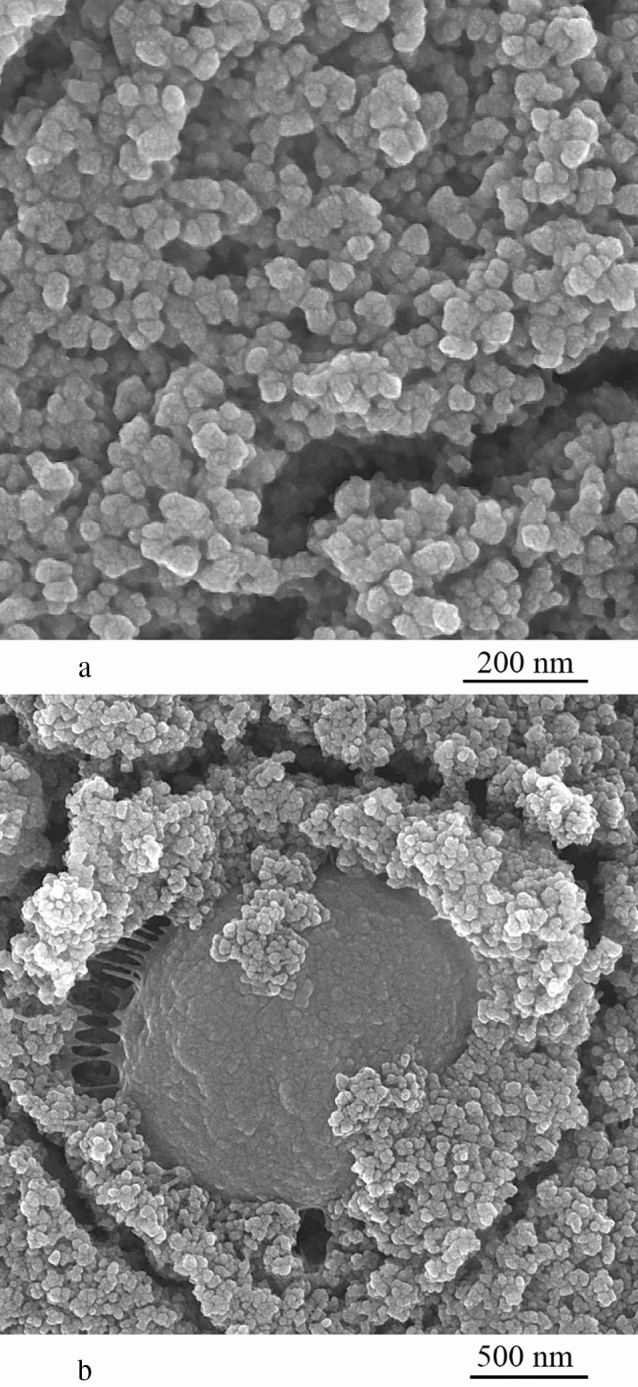
FESEM micrographs of iron nanoparticles (**a**) and iron nanoparticles attached on the surface of a *C. vulgaris* algal cell (**b**)

**Fig. 7:**
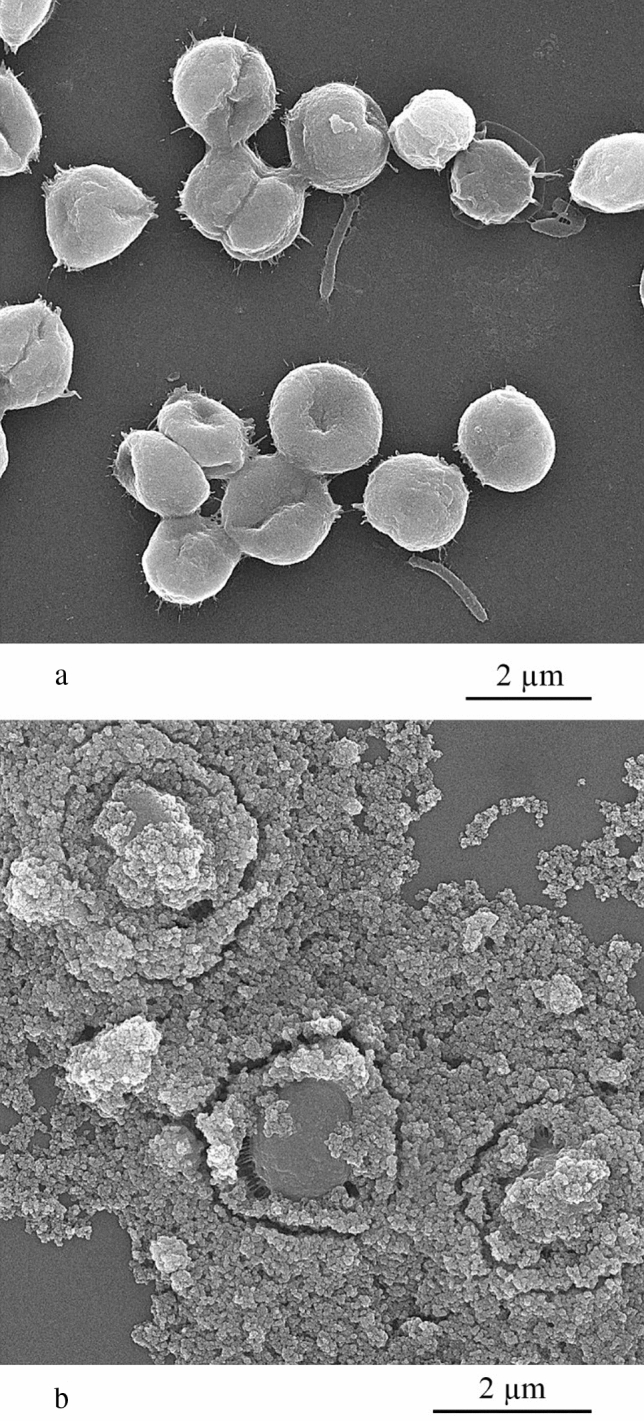
FESEM micrographs of *C. vulgaris* algal cells (**a**) and *C. vulgaris* cells entrapped in aggregations of iron nanoparticles (**b**)

Interaction between microalgal cells and nanoparticles was visualized by FESEM and micrographs as depicted in Fig. 6. It can be observed that lys@IONPs were covering the surface of the microalgal cells. This occurred by non-specific interactions between the nanoparticles and cell envelop that is inline with previous observations reported between IONPs with bacterial cells [33]. In Fig. 7, micrographs for wider view indicated the entrapment of microalgal cells in aggregations of nanoparticles. lys@IONPs tend to settle and aggregate in culture media and interaction of nanoparticles with the cells surface makes them heavier so it may induce the cluster of algal cells and nanoparticles [20, 25, 34, 35].

### Growth Assay

**Fig. 8:**
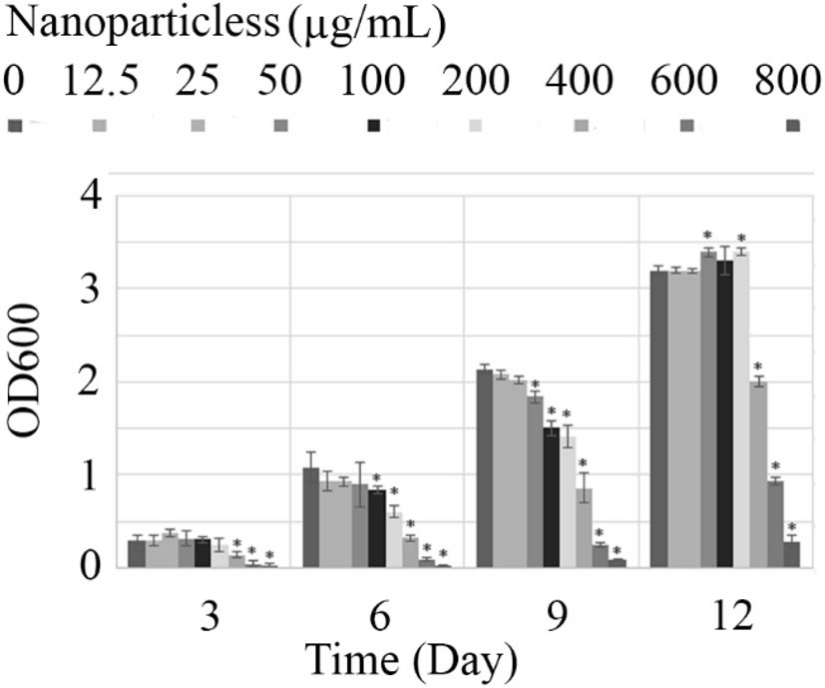
Effects of iron nanoparticles on the growth of *C. vulgaris* cells trough 12-day of exposure. Asterisks indicate significant different (*p*-value ˂ 0.05) with control group (0 concentration of lys@IONPs in each day)

The result of Lys@IONPs impact on the growth of *C. vulgaris* cells that evaluated over 12 days of experiment is depicted in Fig. 8. It was found that iron-based nanoparticles have toxic effects on the microalgal cells. The toxic effect is concentration-dependent and significant reduction in the growth rate can be occurred in the cells that were exposed to the 50 μg/mL of nanoparticles or more concentrations. It is interesting that effect of lys@IONPs on the growth of *C. vulgaris* cells is also dependent to the exposure time. It was found that after 12-day exposure to lys@IONPs the cells can adapt to tolerate up to 200 μg/mL of lys@IONPs. In the case of microalgal cells, similar observation was also reported by direct cell count [20]. But, there are rare reports about other microbial cells exposure with iron oxide nanoparticles for a long period of time. After long-time exposure cells can adapt with toxic materials and recovered cell damages. During exposure time, resistant microalgal cells were selected and became the majority of population. These resistant cells were produced accidentally during cell division by rare spontaneous mutations [36]. It was shown that, similar to microalgal cells, bacterial cells are also sensitive to iron-based nanoparticles in a short exposure time (5-day) [37, 38]. It should be considered that sensitivity of microbial cells to the iron oxide nanoparticles is more related to the microbial strain. For instance, *Pichia pastoris* yeast cells were found to be resistant to iron oxide nanoparticles (up to 200 mg/mL) in a 5-day exposure time [39]. Other yeast strain (*Saccharomyces cerevisiae*) was reported to be resistant up to 500 mg/mL lys@IONPs in a short (overnight) exposure time [40]. Based on results it was demonstrated that low concentration of lys@IONPs has no adverse effect on microalgal growth during long exposure, so it may be considered as safe materials without any adverse effect on aquatic ecosystem.

### Biofilm Assay

**Fig. 9:**
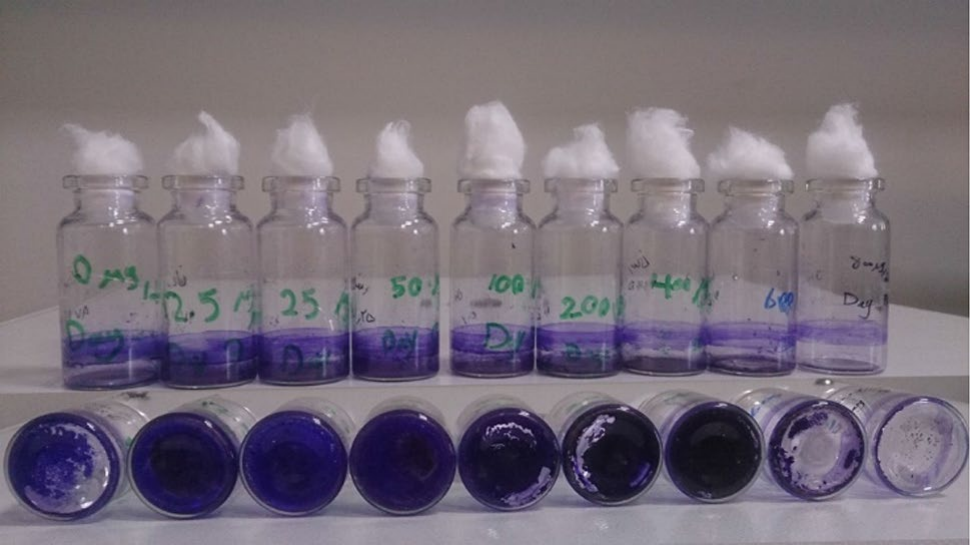
Crystal violet-stained microalgal biofilm on the glass vials, and biofilms were formed in the presence of different concentrations (right to left 0–800 μg/mL) of iron nanoparticles

**Fig. 10:**
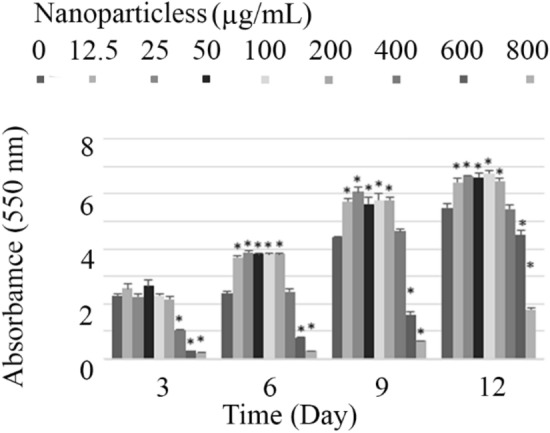
Biofilm formation by microalgal cells which exposed to different concentrations of iron nanoparticles. Asterisks indicate significant different (*p*-value ˂ 0.05) with control group (0 µg/mL lys@IONPs in each day)

Figure 9 illustrates the crystal violet-stained microalgal biofilm on the glass vials. Results showed that biofilm is formed in the absence and also presence of lys@IONPs. As depicted in Fig. 10, exposure to synthesized nanoparticles promotes the biofilm formation in a concentration- and exposure time-dependent manner. Exposure to less than 400 μg/mL of lys@IONPs was resulted in a significant increase in the biofilm formation. However, higher concentrations showed an inhibitory effect on biofilm formation. By increase in the exposure time, adaptation to higher concentrations of lys@IONPs occurred. It was seen that microalgal cells which were exposed to 400 μg/mL of these nanoparticles can overcome the toxic effects of lys@IONPs after 6 days of exposure. Interestingly, after 12 days of exposure, biofilm formation was increased in the presence of 600 μg/mL and 800 μg/mL of lys@IONPs but it is still much lower than the control (in absence of lys@IONPs).

These results illustrate the ability of *C. vulgaris* cells to prevail the toxic effects of iron nanoparticles. It seems that exposure to lys@IONPs induces the cellular adaptation after few days of exposure, *C. vulgaris* cells began to overcome the damages, and the cells began to grow and form biofilm. Also, as illustrated in microscopic micrographs, microalgal cells were entrapped in lys@IONPs and made cell nanoparticle clusters which can promote the biofilm formation. These results are in close agreement with our previous report about the effects of iron nanoparticles on the growth and metabolic activity of *C. vulgaris* [20]. To some extent, a similar pattern was also reported in bacterial cells and differences in details can be due to difference in the examined concentrations and also difference in micro-organisms [11, 41–43]. For instance, it was showed that 10 μg/mL of iron-based nanoparticles can promote biofilm formation in *Pseudomonas aeruginosa*. However, higher concentrations were found to decrease biofilm formation [41]. In another study, presence of 10–100 μg/mL iron nanoparticles inhibited biofilm formation by *S. aureus*, *E. coli*, and *P. aeruginosa* species [43]. Similarly, 100 μg/mL of iron nanoparticles decreased the biofilm formation in *S. epidermidis* [44].

Type of nanoparticles is one of the key features that can modulate the impact of nanoparticles on biofilm formation. lys@IONPs are considered as biocompatible nanoparticles that have no adverse effects on the cell viability and morphology [25]. But, silver and gold nanoparticles are particles that are reported to have anti-biofilm effects even at very low concentrations (under 20 µg/mL) in comparing to iron-based nanoparticles [45, 46]. Zinc oxide, copper oxide, and titanium oxide nanoparticles also showed the inhibitory effect on biofilm formation but at higher concentrations [47]. Concentration-dependent impact of these types of nanoparticles on biofilm formation was reported previously [45, 48]. In the case of silver nanoparticles, it was revealed that low concentration of AgNPs remarkably delayed the biofilm development, whereas high concentration of AgNPs (18 µg/mL) fully stopped the biofilm formation [45].

Coating of nanoparticles can be another effective parameter. It was observed that naked and APTES-coated iron oxide nanoparticles were able to reduce the biofilm formation by *Bacillus subtilis* in 100–400 μg/mL and 300–400 μg/mL, respectively [11]. Based on the results and previous reports it can be concluded that low concentrations of iron nanoparticles promote the growth of microbial cells (microalgae and bacteria) and facilitate the biofilm formation [20, 41].

Different mechanisms were reported for IONPs toxicity, one of the best known mechanisms is release of iron ions due to surface oxidation. These iron ions can catalyze the ROS reactions and produce reactive oxygen species which is toxic to cells and promote damage to cell organelles, such as mitochondria [20]. Also, some investigations realized IONPs toxicity on microalgal cell because of reduction in light availability for photosynthetic processes [50]. Meanwhile, high concentrations of lys@IONPs result in two contradictory effects on the biofilm formation. While the toxic effects of lys@IONPs reduce cells growth, high concentrations of nanoparticles induce cells aggregation and sedimentation.

## Conclusion

*Chlorella vulgaris* was selected as a model of microalgal cells to investigate the effects of lys@IONPs on microalgal biofilm formation. Results revealed that lys@IONPs have concentration- and exposure time-dependent influence on the biofilm formation. Low concentrations (12.5–200 µg/mL) of lys@IONPs increase the cells growth rate and can promote formation of algal biofilm. lys@IONPs at 12.5 µg/mL, therefore, could be an ideal concentration to increase the biofilm formation when higher biofilm formation is needed. On the other hand, exposure to high concentrations (≥ 400 µg/mL) of lys@IONPs would reduce the biofilm formation in a limited period and can be used when low biofilm formation is necessary. Interestingly, in long exposure times, cells can also adapt with high concentrations (≥ 400 µg/mL) to prevail the toxic effects of lys@IONPs. These data indicated that formation of microalgal biofilm is in close relation with the cells growth and population. During exposure to high concentration of lys@IONPs, microalgal cells learn how to overcome this physiological stress and build microbial community. This research emphasizes the feasibility of lys@IONPs to influence the formation of microalgal biofilms for industrial processes by considering its concentration and also exposure time.

## References

[1] Ebrahiminezhad A, Taghizadeh S, Ghasemi Y, Berenjian A (2017). Green synthesized nanoclusters of ultra-small zero valent iron nanoparticles as a novel dye removing material. Science of the Total Environment.

[2] Ranmadugala D, Ebrahiminezhad A, Manley-Harris M, Ghasemi Y, Berenjian A (2017). Iron oxide nanoparticles in modern microbiology and biotechnology. Critical Reviews in Microbiology.

[3] Seifan M, Ebrahiminezhad A, Ghasemi Y, Samani AK, Berenjian A (2017). Amine-modified magnetic iron oxide nanoparticle as a promising carrier for application in bio self-healing concrete. Applied Microbiology and Biotechnology.

[4] Seifan M, Ebrahiminezhad A, Ghasemi Y, Samani AK, Berenjian A (2018). The role of magnetic iron oxide nanoparticles in the bacterially induced calcium carbonate precipitation. Applied Microbiology and Biotechnology.

[5] Seifan M, Sarmah AK, Ebrahiminezhad A, Ghasemi Y, Samani AK, Berenjian A (2018). Bio-reinforced self-healing concrete using magnetic iron oxide nanoparticles. Applied Microbiology and Biotechnology.

[6] Seifan M, Sarmah AK, Samani AK, Ebrahiminezhad A, Ghasemi Y, Berenjian A (2018). Mechanical properties of bio self-healing concrete containing immobilized bacteria with iron oxide nanoparticles. Applied Microbiology and Biotechnology.

[7] Ebrahiminezhad A, Ghasemi Y, Rasoul-Amini S, Barar J, Davaran S (2013). Preparation of novel magnetic fluorescent nanoparticles using amino acids. Colloids and Surfaces B: Biointerfaces.

[8] Ebrahiminezhad A, Rasoul-Amini S, Davaran S, Barar J, Ghasemi Y (2014). Impacts of iron oxide nanoparticles on the invasion power of *Listeria monocytogenes*. Current Nanoscience.

[9] Ebrahiminezhad A, Rasoul-Amini S, Kouhpayeh A, Davaran S, Barar J, Ghasemi Y (2015). Impacts of amine functionalized iron oxide nanoparticles on HepG2 cell line. Current Nanoscience.

[10] Gholami A, Rasoul-Amini S, Ebrahiminezhad A, Abootalebi N, Niroumand U, Ebrahimi N (2016). Magnetic properties and antimicrobial effect of amino and lipoamino acid coated iron oxide nanoparticles. Minerva Biotecnologica.

[11] Ranmadugala D, Ebrahiminezhad A, Manley-Harris M, Ghasemi Y, Berenjian A (2017). The effect of iron oxide nanoparticles on *Bacillus subtilis* biofilm, growth and viability. Process Biochemistry.

[12] Ranmadugala, D., Ebrahiminezhad, A., Manley-Harris, M., Ghasemi, Y., & Berenjian, A. (2017). Impact of 3-aminopropyltriethoxysilane-coated iron oxide nanoparticles on menaquinone-7 production using *B. subtilis*. *Nanomaterials, 7*(11), 350. 10.3390/nano7110350 PMC5707567 29072586

[13] Ranmadugala D, Ebrahiminezhad A, Manley-Harris M, Ghasemi Y, Berenjian A (2017). Magnetic immobilization of bacteria using iron oxide nanoparticles. Biotechnology Letters.

[14] Ansari F, Grigoriev P, Libor S, Tothill IE, Ramsden JJ (2009). DBT degradation enhancement by decorating *Rhodococcus erythropolis* IGST8 with magnetic Fe_3_O_4_ nanoparticles. Biotechnology and Bioengineering.

[15] Aruguete DM, Hochella MF (2010). Bacteria-nanoparticle interactions and their environmental implications. Environmental Chemistry.

[16] Chwalibog A, Sawosz E, Hotowy A, Szeliga J, Mitura S, Mitura K (2010). Visualization of interaction between inorganic nanoparticles and bacteria or fungi. International Journal of Nanomedicine.

[17] Huang YF, Wang YF, Yan XP (2010). Amine-functionalized magnetic nanoparticles for rapid capture and removal of bacterial pathogens. Environmental Science and Technology.

[18] Li YG, Gao HS, Li WL, Xing JM, Liu HZ (2009). In situ magnetic separation and immobilization of dibenzothiophene-desulfurizing bacteria. Bioresource Technology.

[19] Chifiriuc C, Lazar V, Bleotu C, Calugarescu I, Grumezescu AM, Mihaiescu DE (2011). Bacterial adherence to the cellular and inert substrate in the presence of Cofe_2_O_4_ and Fe_3_O_4_/oleic acid-core/shell. Digest Journal of Nanomaterials and Biostructures.

[20] Taghizadeh SM, Berenjian A, Chew KW, Show PL, Mohd Zaid HF, Ramezani H (2020). Impact of magnetic immobilization on the cell physiology of green unicellular algae *Chlorella vulgaris*. Bioengineered.

[21] Ebrahiminezhad A, Bagheri M, Taghizadeh S, Berenjian A, Ghasemi Y (2016). Biomimetic synthesis of silver nanoparticles using microalgal secretory carbohydrates as a novel anticancer and antimicrobial. Advances in Natural Sciences: Nanoscience and Nanotechnology.

[22] Rumbaugh KP, Sauer K (2020). Biofilm dispersion. Nature Reviews Microbiology.

[23] Mantzorou A, Ververidis F (2019). Microalgal biofilms: A further step over current microalgal cultivation techniques. Science of the Total Environment.

[24] Ebrahiminezhad A, Ghasemi Y, Rasoul-Amini S, Barar J, Davaran S (2012). Impact of amino-acid coating on the synthesis and characteristics of iron-oxide nanoparticles (IONs). Bulletin of the Korean Chemical Society.

[25] Shelat R, Bhatt LK, Paunipagar B, Kurian T, Khanna A, Chandra S (2020). Regeneration of hyaline cartilage in osteochondral lesion model using l-lysine magnetic nanoparticles labeled mesenchymal stem cells and their in vivo imaging. Journal of Tissue Engineering and Regenerative Medicine.

[26] Lin S-T, Thirumavalavan M, Lee J-F (2018). A comprehensive study on the mechanism for controlled synthesis of ZnO-based nanomaterials via various polysaccharides as chelates. Results in Physics.

[27] Silva V, Andrade P, Silva M, Valladares LDLS, Aguiar JA (2013). Synthesis and characterization of Fe_3_O_4_ nanoparticles coated with fucan polysaccharides. Journal of Magnetism and Magnetic Materials.

[28] Tan G, Li W, Cheng J, Wang Z, Wei S, Jin Y (2016). Magnetic iron oxide modified pyropheophorbide-a fluorescence nanoparticles as photosensitizers for photodynamic therapy against ovarian cancer (SKOV-3) cells. Photochemical & Photobiological Sciences.

[29] Belachew N, Tadesse A, Kahsay MH, Meshesha DS, Basavaiah K (2021). Synthesis of amino acid functionalized Fe_3_O_4_ nanoparticles for adsorptive removal of rhodamine B. Applied Water Science.

[30] Rodriguez, A. F., Dos Santos, C. C., Lüdtke-Buzug, K., Bakenecker, A. C., Chaves, Y. O., Mariúba, L. A., et al. (2021). Evaluation of antiplasmodial activity and cytotoxicity assays of amino acids functionalized magnetite nanoparticles: Hyperthermia and flow cytometry applications. *Materials Science and Engineering: C, 125*, 112097. 10.1016/j.msec.2021.112097 33965107

[31] Elmaria FA, Jenie SA (2021). Magnetic nanoparticles based on natural silica as a methyl ester forming acid catalyst. Jurnal Kimia Terapan Indonesia.

[32] Chircov C, Matei M-F, Neacșu IA, Vasile BS, Oprea O-C, Croitoru A-M (2021). Iron oxide–silica core–shell nanoparticles functionalized with essential oils for antimicrobial therapies. Antibiotics.

[33] Raee, M. J., Ebrahiminezhad, A., Gholami, A., Ghoshoon, M. B., & Ghasemi, Y. (2018). Magnetic immobilization of recombinant *E. coli* producing extracellular asparaginase: An effective way to intensify downstream process. *Separation Science and Technology, 53*(9), 1397–1404.

[34] Ebrahiminezhad A, Taghizadeh S-M, Ghasemi Y, Berenjian A (2020). Immobilization of cells by magnetic nanoparticles.

[35] Bélteky P, Rónavári A, Igaz N, Szerencsés B, Tóth IY, Pfeiffer I (2019). Silver nanoparticles: Aggregation behavior in biorelevant conditions and its impact on biological activity. International Journal of Nanomedicine.

[36] Ipatova V, Spirkina N, Dmitrieva A (2015). Resistance of microalgae to colloidal silver nanoparticles. Russian Journal of Plant Physiology.

[37] Ebrahiminezhad A, Varma V, Yang S, Berenjian A (2016). Magnetic immobilization of *Bacillus subtilis* natto cells for menaquinone-7 fermentation. Applied Microbiology and Biotechnology.

[38] Ebrahiminezhad A, Varma V, Yang S, Ghasemi Y, Berenjian A (2015). Synthesis and application of amine functionalized iron oxide nanoparticles on menaquinone-7 fermentation: A step towards process intensification. Nanomaterials.

[39] Tagizadeh, S.-M., Ebrahiminezhad, A., Ghoshoon, M. B., Dehshahri, A., Berenjian, A., & Ghasemi, Y. (2021). Impacts of magnetic immobilization on the growth and metabolic status of recombinant *Pichia pastoris*. *Molecular Biotechnology*. 10.1007/s12033-021-00420-w 10.1007/s12033-021-00420-w 34647242

[40] Firoozi, F. R., Raee, M. J., Lal, N., Ebrahiminezhad, A., Teshnizi, S. H., Berenjian, A., et al. (2021). Application of magnetic immboilization for ethanol biosynthesis using *Saccharomyces cerevisiae*. *Separation Science and Technology*. 10.1080/01496395.2021.1939376

[41] Shojaei S, Shojaei S (2019). Optimization of process variables by the application of response surface methodology for dye removal using nanoscale zero-valent iron. International Journal of Environmental Science and Technology.

[42] Ji H, Zhu Y, Duan J, Liu W, Zhao D (2019). Reductive immobilization and long-term remobilization of radioactive pertechnetate using bio-macromolecules stabilized zero valent iron nanoparticles. Chinese Chemical Letters.

[43] Campos, E. A., Pinto, D. V. B. S., de Oliveira, J. I. S., da Costa Mattos, E., & de Cássia Lazzarini Dutra, R. (2015). Synthesis, characterization and applications of iron oxide nanoparticles—A short review. *Journal of Aerospace Technology and Management, 7*(3), 267–276.

[44] Dieudonné A, Pignol D, Prévéral S (2019). Magnetosomes: Biogenic iron nanoparticles produced by environmental bacteria. Applied Microbiology and Biotechnology.

[45] Guo J, Qin S, Wei Y, Liu S, Peng H, Li Q (2019). Silver nanoparticles exert concentration-dependent influences on biofilm development and architecture. Cell Proliferation.

[46] Bhatia E, Banerjee R (2020). Hybrid silver–gold nanoparticles suppress drug resistant polymicrobial biofilm formation and intracellular infection. Journal of Materials Chemistry B.

[47] Ahmed B, Ameen F, Rizvi A, Ali K, Sonbol H, Zaidi A (2020). Destruction of cell topography, morphology, membrane, inhibition of respiration, biofilm formation, and bioactive molecule production by nanoparticles of Ag, ZnO, CuO, TiO_2_, and Al_2_O_3_ toward beneficial soil bacteria. ACS Omega.

[48] Ouyang K, Mortimer M, Holden PA, Cai P, Wu Y, Gao C (2020). Towards a better understanding of *Pseudomonas putida* biofilm formation in the presence of ZnO nanoparticles (NPs): Role of NP concentration. Environment International.

